# Supplementary material for: End Sequence Analysis Toolkit (ESAT) expands the extractable information from single-cell RNA-seq data
Source: Genome Res. 2016 Oct;26(10):1397–410. doi: 10.1101/gr.207902.116 (PMC5052061; doi:10.1101/gr.207902.116)
Supplement: Supplemental Material [file supp_gr.207902.116_Supp_Materials.tar.gz › ESAT-esat_v0.1/doc/html/classbroad_1_1pda_1_1seq_1_1rap_1_1_skellam_score.html]

ESAT: broad.pda.seq.rap.SkellamScore Class Reference


ESAT


- Main Page
- Related Pages
- Packages
- Classes
- Files

- Class List
- Class Index
- Class Hierarchy
- Class Members

All Classes Namespaces Files Functions Variables Enumerator Pages

- broad
- pda
- seq
- rap
- SkellamScore

Classes |
Public Member Functions |
List of all members

broad.pda.seq.rap.SkellamScore Class Reference

Inheritance diagram for broad.pda.seq.rap.SkellamScore:

[legend]

Collaboration diagram for broad.pda.seq.rap.SkellamScore:

[legend]

|  |  |
| --- | --- |
| Classes | |
| class | **Processor** |
|  | |

|  |  |
| --- | --- |
| Public Member Functions | |
|  | SkellamScore (Annotation a) |
|  | |
|  | SkellamScore (AlignmentModel numerator, AlignmentModel denominator, Annotation annotation, double rapLambda, double backgroundLambda) |
|  | |
| double | getScore () |
|  | |
| double | getNumeratorScanPVal () |
|  | |
| double | getDenominatorScanPVal () |
|  | |
| String | toString () |
|  | |
| double | getSkellamPValue () |
|  | |
| boolean | countLessThanLambda () |
|  | |

## Constructor & Destructor Documentation

|  |  |  |  |  |  |
| --- | --- | --- | --- | --- | --- |
| broad.pda.seq.rap.SkellamScore.SkellamScore | ( | Annotation | *a* | ) |  |

|  |  |  |  |
| --- | --- | --- | --- |
| broad.pda.seq.rap.SkellamScore.SkellamScore | ( | AlignmentModel | *numerator*, |
|  |  | AlignmentModel | *denominator*, |
|  |  | Annotation | *annotation*, |
|  |  | double | *rapLambda*, |
|  |  | double | *backgroundLambda* |
|  | ) |  |  |

Here is the call graph for this function:

## Member Function Documentation

|  |  |  |  |  |
| --- | --- | --- | --- | --- |
| boolean broad.pda.seq.rap.SkellamScore.countLessThanLambda | ( |  | ) |  |

Returns true if the signal count is less than the chromosome lambda

Returns

|  |  |  |  |  |
| --- | --- | --- | --- | --- |
| double broad.pda.seq.rap.SkellamScore.getDenominatorScanPVal | ( |  | ) |  |

|  |  |  |  |  |
| --- | --- | --- | --- | --- |
| double broad.pda.seq.rap.SkellamScore.getNumeratorScanPVal | ( |  | ) |  |

Here is the caller graph for this function:

|  |  |  |  |  |
| --- | --- | --- | --- | --- |
| double broad.pda.seq.rap.SkellamScore.getScore | ( |  | ) |  |

Here is the call graph for this function:

Here is the caller graph for this function:

|  |  |  |  |  |
| --- | --- | --- | --- | --- |
| double broad.pda.seq.rap.SkellamScore.getSkellamPValue | ( |  | ) |  |

Based on prussell's function in PairedSampleCoverage Compute Skellam P-value of read counts in a region given the two parameters denominatorLambda Poisson lambda for background sample numeratorLambda Poisson lambda for signal sample denominatorCount The count for background sample numeratorCount The count for signal sample

Returns
:   The probability under the null hypothesis of observing a greater difference

Exceptions
:   |  |  |
    | --- | --- |
    | IOException |  |

Here is the caller graph for this function:

|  |  |  |  |  |
| --- | --- | --- | --- | --- |
| String broad.pda.seq.rap.SkellamScore.toString | ( |  | ) |  |

Here is the call graph for this function:

---

The documentation for this class was generated from the following file:

- java/broad/pda/seq/rap/SkellamScore.java


---

Generated on Fri Aug 15 2014 15:11:03 for ESAT by  

 1.8.7
